# Automated detection of early signs of irreversible ischemic change on CTA source images in patients with large vessel occlusion

**DOI:** 10.1371/journal.pone.0304962

**Published:** 2024-06-13

**Authors:** Adrian Mak, Charles C. Matouk, Emily W. Avery, Jonas Behland, Stefan P. Haider, Dietmar Frey, Vince I. Madai, Peter Vajkoczy, Christoph J. Griessenauer, Ramin Zand, Philipp Hendrix, Vida Abedi, Pina C. Sanelli, Guido J. Falcone, Nils Petersen, Lauren H. Sansing, Kevin N. Sheth, Seyedmehdi Payabvash, Ajay Malhotra

**Affiliations:** 1 Department of Radiology and Biomedical Imaging, Section of Neuroradiology, Yale School of Medicine, New Haven, CT, United States of America; 2 CLAIM—Charité Lab for Artificial Intelligence in Medicine, Charité Universitätsmedizin Berlin, Berlin, Germany; 3 Department of Neurosurgery, Division of Neurovascular Surgery, Yale University School of Medicine, New Haven, CT, United States of America; 4 Department of Otorhinolaryngology, LMU Clinic of Ludwig-Maximilians-University of Munich, Munich, Germany; 5 QUEST Center for Responsible Research, Berlin Institute of Health (BIH), Charité Universitätsmedizin Berlin, Berlin, Germany; 6 School of Computing and Digital Technology, Faculty of Computing, Engineering and the Built Environment, Birmingham City University, Birmingham, United Kingdom; 7 Department of Neurosurgery, Charité Universitätsmedizin Berlin, Berlin, Germany; 8 Research Institute of Neurointervention, Paracelsus Medical University, Salzburg, Austria; 9 Department of Neurosurgery, Paracelsus Medical University, Salzburg, Austria; 10 Department of Neurology, Geisinger Medical Center, Danville, PA, United States of America; 11 Department of Neurology, Pennsylvania State University, State College, PA, United States of America; 12 Department of Neurosurgery, Geisinger Medical Center, Danville, PA, United States of America; 13 Department of Neurosurgery, Saarland University Medical Center, Homburg, Germany; 14 Department of Public Health Sciences, The Pennsylvania State University, Hershey, PA, United States of America; 15 Department of Molecular and Functional Genomics, Geisinger Medical Center, Danville, PA, United States of America; 16 Department of Radiology, Northwell Health Feinstein Institutes for Medical Research, Manhasset, New York, United States of America; 17 Division of Neurocritical Care and Emergency Neurology, Department of Neurology, Yale University School of Medicine, New Haven, CT, United States of America; 18 Division of Stroke and Vascular Neurology, Department of Neurology, Yale University School of Medicine, New Haven, CT, United States of America; Stanford University School of Medicine, UNITED STATES

## Abstract

**Purpose:**

To create and validate an automated pipeline for detection of early signs of irreversible ischemic change from admission CTA in patients with large vessel occlusion (LVO) stroke.

**Methods:**

We retrospectively included 368 patients for training and 143 for external validation. All patients had anterior circulation LVO stroke, endovascular therapy with successful reperfusion, and follow-up diffusion-weighted imaging (DWI). We devised a pipeline to automatically segment Alberta Stroke Program Early CT Score (ASPECTS) regions and extracted their relative Hounsfield unit (rHU) values. We determined the optimal rHU cut points for prediction of final infarction in each ASPECT region, performed 10-fold cross-validation in the training set, and measured the performance via external validation in patients from another institute. We compared the model with an expert neuroradiologist for prediction of final infarct volume and poor functional outcome.

**Results:**

We achieved a mean area under the receiver operating characteristic curve (AUC), accuracy, sensitivity, and specificity of 0.69±0.13, 0.69±0.09, 0.61±0.23, and 0.72±0.11 across all regions and folds in cross-validation. In the external validation cohort, we achieved a median [interquartile] AUC, accuracy, sensitivity, and specificity of 0.71 [0.68–0.72], 0.70 [0.68–0.73], 0.55 [0.50–0.63], and 0.74 [0.73–0.77], respectively. The rHU-based ASPECTS showed significant correlation with DWI-based ASPECTS (r_S_ = 0.39, p<0.001) and final infarct volume (r_S_ = -0.36, p<0.001). The AUC for predicting poor functional outcome was 0.66 (95%CI: 0.57–0.75). The predictive capabilities of rHU-based ASPECTS were not significantly different from the neuroradiologist’s visual ASPECTS for either final infarct volume or functional outcome.

**Conclusions:**

Our study demonstrates the feasibility of an automated pipeline and predictive model based on relative HU attenuation of ASPECTS regions on baseline CTA and its non-inferior performance in predicting final infarction on post-stroke DWI compared to an expert human reader.

## Introduction

The Alberta Stroke Program Early CT Score (ASPECTS) has been widely applied for semiquantitative assessment of early signs of irreversible ischemic injury in the middle cerebral artery (MCA) territory over the past two decades [1]. ASPECTS was originally proposed to identify patients with large ischemic infarct volume on baseline non-contrast computed tomography (NCCT), as potential exclusion criteria for intravenous thrombolytic therapy [2]. Its use has since been extended to identifying patients for treatment with endovascular thrombectomy in trials and clinical practice [3]. Eligibility screening for thrombectomy using ASPECTS on admission non-contrast CT is however debated, with recent findings implicating improved functional outcome in patients with large ischemic strokes even below the established threshold of ASPECTS > 6 [4].With the opportunity of being obtained with minimal delay after NCCT and as an integral imaging component of the LVO diagnostic workflow, CT angiography (CTA) source images provide a valuable additional modality for detecting ischemic injury. Since hypo-attenuation on CTA source images correlates with depressed cerebral blood flow, rather than edema formation as on NCCT, the ASPECTS on CTA source images has been shown to be more sensitive in the detection of early irreversible ischemia and more accurate in the prediction of final infarct volume [5–7]. ASPECTS quantification on CTA source images in acute ischemic stroke workup provides an enhanced volumetric estimate of irreversible ischemic damage and can potentially improve treatment selection.

Nevertheless, visual quantification of early infarction can be time-consuming and prone to inter-observer variability [8–10]. Given the importance of ASPECTS in patient selection for thrombolysis and/or thrombectomy as well as for inclusion in clinical trials, automated evaluation of ASPECTS can provide an appealing alternative to visual assessment. It can expedite and improve the objectivity of the treatment triage process [11]. Few commercially available software packages for automated quantification of ASPECTS have recently been evaluated on NCCT [12–14]. These applications are, however, not validated for usage on CTA and prior models for detection of hypo-attenuation and evaluation of ASPECTS on CTA source images lack independent validation [15] or reliable ground-truth labeling [16]. The generalizability of these findings across institutions and their potential clinical relevance of their predictions remains unclear.

Therefore, we aimed to develop a proof-of-concept automated pipeline for evaluation of early signs of irreversible ischemic change on admission CTA source images of acute LVO stroke patients and measured its performance in cross-validation as well as in an external validation cohort from another institution. Post-treatment diffusion-weighted magnetic resonance imaging (DWI) after successful endovascular thrombectomy was used to determine the final infarct lesions. To assess clinical relevance, we compared the performance of our automated ASPECTS evaluation with visually quantified ASPECTS in predictions of DWI-based ASPECTS, final infarct volume, and poor functional outcome.

## Methods

### Study design and data collection

A total of 368 acute stroke patients from the Yale New Haven Hospital were included as training cohort for our model. All of these patients were part of the prospective Stroke Biorepository at the institute and were admitted between January 2014 and October 2020. We validated our model in an external cohort of 143 patients from the Geisinger Institute (admitted from January 2016 to December 2019), following the same inclusion and exclusion criteria. Patients’ data was accessed between November 21^th^ 2018 and April 1^st^ 2021. The investigators had access to identifying patient information during the initial collection, which was then pseudonymized for all later analyzes. The inclusion criteria were: (1) evidence of unilateral anterior circulation LVO on admission CTA, defined as occlusion of the internal carotid artery (ICA) and/or middle cerebral artery (M1 or proximal M2 segments); (2) endovascular mechanical thrombectomy with successful reperfusion, i.e. modified Treatment In Cerebral Ischemia (mTICI) score ≥ 2b; and (3) availability of post-stroke MRI. Patients were excluded if the quality of either CTA source images or MRI scans prohibited image analysis, their functional outcome wasn’t documented, or they had a stroke 3 months before or immediately after the index endovascular therapy. Functional outcome was determined using the modified Rankin Scale (mRS) 3 months after discharge, defining poor outcome as mRS >2. The collateral status of patients was evaluated by two blinded neuroradiologist readers on the 3-point rating scale proposed by Miteff et al. [17] using consensus rating. When 3 months mRS was not available, discharge mRS was imputed. This study was approved by Yale’s Institutional Review Board (#2000024296). Informed consent was waived given the minimal risk to subjects.

### Imaging protocol and endovascular thrombectomy

All decisions pertaining the treatment of patients, including the choice of device for mechanical thrombectomy and timing of post-intervention imaging, were made by the patients’ stroke care teams in accordance with the AHA/ASA guidelines and institutional protocols at time of admission. CT angiographic acquisition at admission was performed following non-contrast CT and according to standard departmental protocols, mainly using Revolution CT scanners (GE Healthcare). The parameters mainly used were: 100 kVp, 200 mAs, and 0.625mm axial slice thickness. After the intravenous power injection of 70 mL non-ionic contrast material, serial axial thin sections were obtained from the aortic arch to the vertex. Endovascular thrombectomy was performed when an occlusion of the ICA, M1 or M2 was detected in admission CTA as per then-current treatment guidelines. Endovascular thrombectomy was performed predominantly combining stent retrievers and aspiration catheters or using aspiration catheters only. The modified Thrombolysis in Cerebral Infarction (mTICI) score documented in the official clinical report of the endovascular intervention was used to identify patients with successful reperfusion (mTICI 2b or 3). Post-stroke imaging consisted of diffusion-weighted imaging (DWI) for all patients, acquired between 24 hours up to 7 days after the intervention.

### Visual assessment of ASPECTS on CTA source images

A neuroradiologist reader (S.P.) with over 10 years of experience, blinded to patients’ characteristics and outcomes, determined overall visual ASPECTS on CTA source images in the validation cohort.

### Automated measurement of final infarction on post-treatment DWI

**Fig 1:**
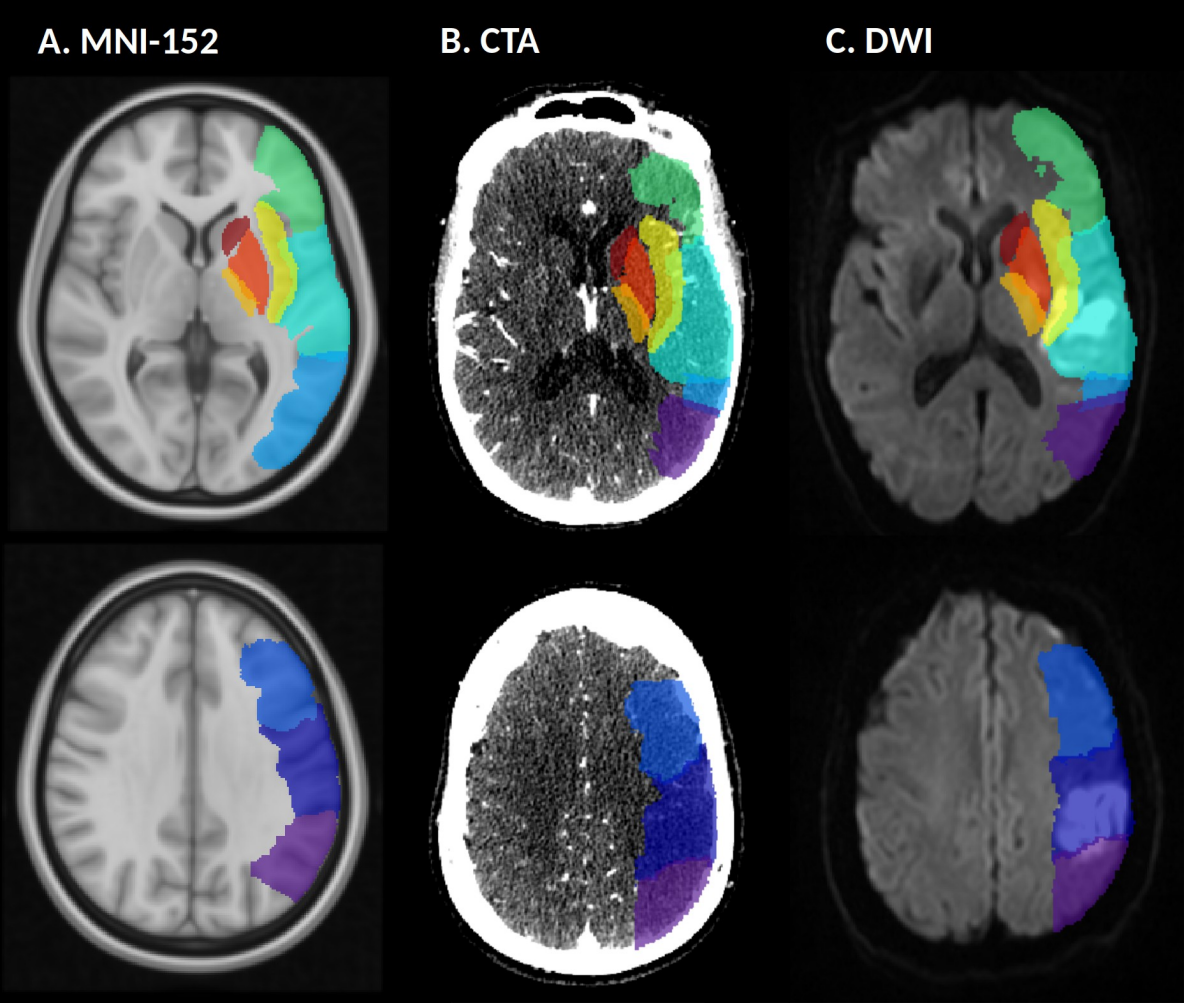
Representation of the ASPECTS regions. Illustration of the 10 ASPECTS 3D-masks of the left hemisphere in (A) MNI-152 space, (B) coregistered to a CTA source image, and (C) coregistered to a post-stroke DWI. Each color represents one of the ASPECTS regions.

Adapting from the anatomical Harvard-Oxford atlas, we generated segmentations of all ASPECTS regions in standard MNI-152 space [18]. The resulting atlas contained ten 3-dimensional masks per hemisphere, each representing one of the ASPECTS regions (Fig 1). We manually segmented the post-intervention infarct lesions on follow-up DWI, using MRIcro image viewer. The infarct lesions were saved as binary masks and measured for final infarct volume using FSLUTILS. To dichotomize each region as infarcted or non-infarcted for each patient, we coregistered each ASPECTS region mask onto the patient’s native DWI using FMRIB’s Linear Image Registration Tool [19] and calculated the intersection with the final infarct lesion mask. We defined a region as infarcted when ≥30% of its total voxels were intersecting.

### Automated measurement of relative HU intensity on CTA source images

The ASPECTS region masks were coregistered to each patient’s CTA scan, using the reverse matrix created by coregistering each CTA to MNI-152 space and FMRIB’s Linear Image Registration Tool. We calculated the relative signal intensity in Hounsfield Units (rHU) of each ASPECTS region by dividing the mean attenuation of the region on the occlusion side by the mean average attenuation of the corresponding contralateral region on the non-occluded side. To compensate for potential inaccuracy of the coregistration process and differences in the intravenous bolus timing among different CTA scans, only voxels within the range of 1–250 Hounsfield units were included for calculating the mean attenuation.

### Optimal relative HU cut points for threshold-based prediction of final infarction

We used the training set of 368 cases to determine the cut point of rHU with the highest Youden-Index for the prediction of final infarction in each ASPECTS region. We then applied these optimal cut points to the validation set of 143 cases, predicting a region to be finally infarcted when its rHU value was below the respective optimal threshold for that region. The receiver operating characteristics area under curve (AUC), accuracy, sensitivity, and specificity were calculated as diagnostic metrics for each region. Single round k-fold cross-validation (k = 10) with random allocation of cases was performed within the training set to measure model stability. For each fold, the optimal cut point was determined in nine folds and applied to the left-out fold for prediction. We also calculated Spearman’s correlation coefficient r_S_ with the final DWI-ASPECTS and final infarct volume for the validation cohort. We compared the r_S_ of threshold-based ASPECTS and visual ASPECTS using R. R. Wilcox’s percentile bootstrap method [20]. AUCs for the prediction of poor functional outcome were calculated for both ASPECTS and compared using DeLong’s test via the pROC-package [21].

### Statistical analysis and code

The results are presented as mean ± standard deviation, median [interquartile range], or number (frequency), wherever appropriate. We applied Student’s t-test, *Χ^2^* test, and Mann-Whitney U for univariate analysis. All statistical analyses were performed using R Version 4.3 and R Studio Version 1.4 (RStudio PBC, Boston, USA). The code for our automated segmentation pipeline, the 3D masks of the ASPECTS regions, and an implementation of the rHU prediction model can be found here: https://github.com/AnotherMak/aspects-on-cta

## Results

### Patients’ characteristics

Table 1 summarizes the demographic and clinical characteristics of the training (n = 368) and independent validation (n = 143) cohorts separately. Patients in our validation cohort had higher NIHSS at admission (14 [9 – 19] versus 17 [12 – 22], p <0.001) and fewer M2 occlusions (34.0% versus 14.0%, p<0.001). The validation cohort had a higher proportion of patients with mTICI = 3, i.e. complete antegrade reperfusion after thrombectomy (29.9% versus 63.6%, <0.001). There were no differences in patient outcomes such as final infarct volume (44.0±62.4 versus 52.2±76.4, p = 0.21), modified Rankin Scale score at 3-month follow up (3 [1 – 4] versus 3 [1 – 4], p = 0.40), mortality at 3-month follow up (13.9% versus 14.7%, p = 0.92) or poor functional outcome (57.9% versus 55.9%, p = 0.64). The types of endovascular interventions performed in both cohorts can be found in the supporting information (S1 File).

**Table 1:** Demographic and clinical characteristics of the study cohort.

	Training (n = 368)	Validation (n = 143)	p-values
Age (years)	68.0±15.4	68.5±14.9	0.75
Male sex	189 (51.4%)	65 (45.5%)	0.27
Right sided LVO	166 (45.1%)	71 (49.7%)	0.41
Admission NIHSS score	14 [9–19]	17 [12–22]	**<0.001**
Onset-to-catheterization (hours)	7.3±5.4	6.5±4.7	0.13
Occlusions			
ICA occlusion	73 (19.8%)	28 (19.6%)	1.00
MCA M1 occlusion	230 (62.5%)	94 (65.7%)	0.56
MCA M2 occlusion	125 (34.0%)	20 (14.0%)	**<0.001**
Collateral status			0.60
Good	162 (44%)	58 (40.6%)	
Moderate	135 (36.7%)	52 (36.4%)	
Poor	71 (19.3%)	33 (23.1%)	
Intravenous thrombolysis	163 (44.3%)	62 (43.4%)	0.93
mTICI 3	110 (29.9%)	91 (63.6%)	**<0.001**
Hemorrhagic transformation	182 (49.5%)	57 (39.9%)	0.06
Symptomatic hemorrhage *	21 (5.7%)	8 (5.6%)	0.93
3-month mRS score	3 [1–4]	3 [1–4]	0.40
3-month mortality	51 (13.9%)	21 (14.7%)	0.92
Poor functional outcome†	216 (58.7%)	80 (55.9%)	0.64
Final infarct lesion volume (mL)	44.0±62.4	52.2±76.4	0.21

Patients’ characteristics at admission and discharge for the training and validation set. Significant p-values are in bold.

* Symptomatic hemorrhage defined by an associated increase of ≥4 points in NIHSS, need for decompressive hemicraniectomy or hemorrhage leading to death

† Poor functional outcome defined as mRS score at 3-month follow up >2

### Threshold-based prediction of final infarction

In ten-fold cross-validation in the training set, we achieved mean AUC, accuracy, sensitivity, and specificity of 0.69±0.13, 0.69±0.09, 0.61±0.23, and 0.72±0.11 across all regions and folds. Table 2 shows the optimal rHU cut point for each ASPECTS region when trained on the entire training set and its performance in predicting final infarction in the validation set. The optimal cut points ranged from 0.950 (M3) to 0.859 (M2). In the validation cohort, the AUC ranged from 0.77 in the lentiform nucleus to 0.64 in the M1 and M4 regions with a median of 0.71 [0.68–0.72]. The accuracy ranged from 0.8 in the M6 region to 0.66 in the M4 region with a median of 0.70 [0.68–0.73]. The sensitivity of the rHU cut points ranged from 0.66 in the lentiform nucleus to 0.42 in the M6 region, with a median of 0.55 [0.50–0.63], while the specificity ranged from 0.85 in the M6 region to 0.69 in the M4 region with a median of 0.74 [0.73–0.77].

**Table 2:** Performance of optimal relative Hounsfield Units (rHU) cut points.

Region	Optimal rHU cut point	AUC	Accuracy	Sensitivity	Specificity	Patients with final infarction
**Caudate**	0.916	0.71	0.69	0.63	0.73	51 (36%)
**Insular Cortex**	0.871	0.72	0.68	0.58	0.75	62 (43%)
**Internal Capsular**	0.949	0.72	0.74	0.64	0.78	36 (25%)
**Lentiform nucleus**	0.930	0.77	0.70	0.66	0.73	62 (43%)
**M1**	0.936	0.64	0.69	0.64	0.70	22 (15%)
**M2**	0.859	0.71	0.68	0.51	0.76	45 (31%)
**M3**	0.950	0.67	0.70	0.50	0.73	18 (12%)
**M4**	0.957	0.64	0.66	0.52	0.69	19 (13%)
**M5**	0.904	0.72	0.75	0.43	0.81	23 (16%)
**M6**	0.920	0.70	0.80	0.42	0.85	19 (13%)

Performance of the optimal relative Hounsfield Units (rHU) cut points in the detection of signs of irreversible ischemic change for patients in the external validation cohort (n = 143) for each ASPECTS region.

### Prediction of tissue and clinical outcomes

Threshold-based ASPECTS had a significant correlation with DWI-ASPECTS (r_S_ = 0.39, 95%CI: 0.24–0.52, p<0.001) and final infarct volume (r_S_ = -0.36, 95%CI: -0.49 –-0.20, p<0.001). There was no significant difference between threshold-based versus visual ASPECTS correlation with DWI-ASPECTS (p = 0.84) and final infarct volume (p = 0.75) (Table 3). Regarding functional outcome, threshold-based ASPECTS showed an AUC of 0.66 (95%CI: 0.57–0.75) for the prediction of poor functional outcome compared to an AUC of 0.60 (95%CI: 0.51–0.69) for visual ASPECTS. The difference was not statistically significant (p = 0.21).

**Table 3:** Comparison of predictive capabilities of visual ASPECTS and threshold-based ASPECTS.

	Visual ASPECTS	Threshold-based ASPECTS	p for comparison
**DWI-ASPECTS**	0.40 (0.25–0.53)	0.39 (0.24–0.52)	0.84
**Final infarct volume**	-0.38 (-0.51 –-0.23)	-0.36 (-0.49 –-0.20)	0.75

Comparison of Spearman’s correlation coefficient r_S_ (95% CI) of visual ASPECTS and threshold-based ASPECTS in the external validation cohort (n = 143).

## Discussion

In this study, we developed an automated pipeline for the segmentation of ASPECTS regions on CTA source images and detection of early signs of irreversible ischemic tissue damage. We calculated the optimal region-specific rHU threshold for prediction of final infarction on post-stroke DWI after successful endovascular thrombectomy in each ASPECTS region. We then used these thresholds to rate the ASPECTS in an external validation cohort from another institution, achieving a performance similar to an experienced neuroradiologist reader in the prediction of radiological and functional outcomes. The predictive capabilities of both ASPECTS ratings regarding clinical outcome were limited, potentially highlighting the difficulty of outcome prediction from admission scans alone. Serving as a proof-of-concept, our automated ASPECTS segmentation pipeline as well as prediction model are publicly available to other investigators with stable performance metrics across cohorts from multiple institutions.

Since CTA scans are an integral part of the LVO diagnostic workflow, CTA source images are available in almost all patients with suspected LVO and can be used for additional prognostication without delaying treatment decisions. Prior studies found more sensitive detection of early signs of irreversible infarction and more accurate assessment of infarct core when comparing CTA source images to NCCT [5, 7]. This is likely because the areas of hypo-attenuation on CTA are more strongly correlated with depressed cerebral blood flow (CBF) rather than volume (CBV) in arterial phase images obtained with current multidetector scanners [22, 23]. However, given prior evidence of inclusion of ischemic penumbra in parenchymal abnormalities as seen on CTA source images, a differentiation between irreversible damage and salvageable penumbra is important for potential clinical applications [24]. By training the model on post-treatment lesion segmentations on DWI after successful endovascular thrombectomy, we intended to limit our signal specifically to signs of irreversible ischemic damage on admission. The cutoff is optimized to exclude regions showing potential parenchymal hypoattenuation caused by salvageable penumbra, since these regions would not evolve into final infarct lesion after the successful intervention. The evaluation of admission CTA source images in a cohort receiving endovascular treatment poses a more complex task than in an untreated cohort, since in the latter case all regions with any signs of hypoattenuation would likely infarct. The differentiation of early signs of irreversible ischemic damage from potentially salvageable penumbra is however of particular relevance, especially in cases where perfusion imaging might not be readily available. To our knowledge, this is the first automated ASPECTS evaluation on CTA source images specifically developed and validated in a cohort of patients treated with endovascular thrombectomy.

There have been previous attempts on implementing automated detection models for ASPECTS on CTA source images in untreated patient cohorts. Reidler et al. [15] performed a software-based segmentation and measurement of rHU in all ASPECTS regions. Analogue to our analysis, they used Youden-Index-derived cut-off values of rHU to predict regional final infarction and achieved AUCs between 0.87 for the caudate and 0.60 for the M5 region. Their model achieved a Pearson Correlation Coefficient of -0.29 (95% CI -0.50 –-0.04) with final infarct volume. However, their study was limited by a small study cohort of 79 patients, without any independent or external validation, which might have resulted in overfitting and overestimation of model accuracy. Their labeling process of final infarction was also partly based on follow-up NCCT instead of DWI. Öman et al [16] deployed a 3D CNN to detect and segment hypoattenuation on CTA source images, comparing their results on region-level against the ASPECTS scoring of two human raters. They used a cohort of 60 patients, 30 of whom had been diagnosed with acute ischemic stroke. Training three separate models with slightly varied inputs, they reported overall sensitivity between 0.71 and 0.67 and overall specificity between 0.96 and 0.93 at a probability threshold of 0.5. Notably, an AUC between 0.91 and 0.93 for detection of infarction was reported. Their study, however, has a few caveats: First, the ground truth of their study was manual segmentations of the same CTA scans that were presented to the CNNs for training and testing. The lack of delayed second imaging or use of another imaging modality for ground truth limits the clinical significance of the model’s prediction since it remains unclear to which extent the human readers and model picked up ischemic core and/or penumbra in their segmentations. Finally, their small sample size of only 30 patients in training and validation each as well as lack of external validation severely limit the generalizability of their findings.

We tried to improve on these limitations by using a large training dataset, validating the performance of our model via cross-validation as well as a dedicated external validation cohort from another institution. Our regional performance was comparable to the prior models, while achieving better correlation to the final infarct volume. The stable performance of the pipeline across multiple institutions gives us confidence that the pipeline sufficiently accommodates for potential inaccuracies in the co-registration process and other confounders. Furthermore, we tried to establish clinical applicability of our model by determining its predictive performance for radiological and functional outcomes. We found the correlation of our model’s threshold-based ASPECTS non-inferior to that of a human reader regarding DWI-ASPECTS as well as final infarct volume. This further strengthens the notion that our model is specifically limiting its signal to hypoattenuation that corresponds to irreversible ischemic change.

The prediction of the long-term clinical outcome of acute ischemic stroke patients after thrombectomy remains a complex challenge. Despite its widespread adaptation after the 2015/2018 landmark trials and its overwhelming efficacy, up to half of all LVO patients suffer severe disability or death even after successful reperfusion [25]. Given the established correlation between hypoattenuation on CTA source images and final infarct volume, ASPECTS on CTA could potentially help identify patients with a large infarct core at admission and resulting high risk of hemorrhagic transformation after intervention to more reliably [26]. Improved patient stratification at admission, guided by complementary information with regard to the likely benefit or harm of treatment, could help to guide treatment decisions and consequently improve patient outcomes [25, 27]. Widely accepted independent predictors for poor long-term outcome are clinical variables such as older age or higher baseline NIHSS, but also extensive infarct demarcation on admission, signified by lower ASPECTS on both NCCT as well as CTA source images [6, 28]. The ASPECTS evaluated by human visual reading and the threshold-based model in our study could predict poor functional patient outcome at the 3 month follow-up with an AUC of 0.60 and 0.66, respectively. While ASPECTS on CTA has been shown to be more predictive of functional outcome than its NCCT counterpart, the weak predictive power regarding long-term outcome we found could highlight an inherent limit of this semiquantitative scoring system when used in isolation from other predictor variables. Proposed AI-based models for clinical outcome prediction in stroke show best performance when using a combined approach, including imaging information from admission CTA source images or other imaging modalities, as well as clinical variables [29, 30]. Our automated pipeline for segmentation and quantification of rHU and threshold-based calculation of ASPECTS is made publicly available, which can facilitate the multicentric evaluation and eventually implementation of our threshold-based approach in more comprehensive models.

Our study has several limitations. First, the date of admission of the patients of both cohorts spans over several years due to the large sample size, potentially increasing the variability of patients by changes in thrombectomy techniques and the expansion of the thrombectomy time-window. Second, the concept of ASPECTS lacks a concrete definition of its regions. While the original ASPECTS allots 3 subcortical (caudate, internal capsular, lentiform nucleus) and 7 cortical points (insular cortex, M1-M6), it does not provide clear boundaries for these areas. This proves a problem for reproducibility and inter-rater concordance for human readers and automated approaches alike, representing an inherent limitation of the ASPECTS concept. We tried to increase the reproducibility of our results by making the 3D masks we used publicly available. Third, CTA source images are at risk of higher inter-patient variability due to differences in the timing of administration of contrast bolus and image acquisition. We attempted to mitigate that risk by limiting our measurements to a range of 1–250 HU and calculating the relative HU for each region, normalizing the measurements in the process. Finally, we did not compare our model’s performance on CTA source images to other imaging modalities such as CTP due to the lack of availability in our study cohort. Future studies comparing the predictive capabilities of hypoattenuation on CTA source images with CTP are warranted.

## Conclusions

Our study demonstrates the feasibility and performance of an automated prediction model based on relative HU attenuation of ASPECTS regions on baseline CTA, aiming to predict final infarction on post-stroke DWI. Cross-validation and external validation suggest stability and transferability of our model across institutions. Its predictive performance was comparable to that of a human neuroradiologist reader for all tested outcomes. Our findings demonstrate the possibility for the development of clinical applications for automated detection of early signs of irreversible ischemic damage on CTA source images and can be built upon in more comprehensive models for acute stroke treatment triage.

## Supporting information

S1 Data Raw data of measurements derived from CTA and DWI in the training and validation dataset. (XLSX)

S1 File Endovascular Therapies. Types of endovascular therapies performed in the training and validation dataset.
(DOCX)
