# Supplementary material for: Automated detection of early signs of irreversible ischemic change on CTA source images in patients with large vessel occlusion
Source: PLoS One. 2024 Jun 13;19(6):e0304962. doi: 10.1371/journal.pone.0304962 (PMC11175522; doi:10.1371/journal.pone.0304962)
Supplement: S1 File — Types of endovascular therapies performed in the training and validation dataset. (DOCX) [file pone.0304962.s002.docx]

Supplemental Table 1. Types of endovascular therapies performed in the training and validation cohorts.

|  | **Training (n=368)** | **Validation (n=143)** | **p-values** |
| --- | --- | --- | --- |
| Stent retriever + Aspiration | 289 (82.8%) | 112 (78.3%) | 0.30 |
| Aspiration only | 60 (17.2%) | 27 (18.9%) | 0.75 |
| Stent retriever only | 0 (0%) | 4 (2.8%) | **0.01** |
| Intra-arterial lysis | 7 (1.9%) | 0 (0%) | 0.22 |

Results are presented as n (%).
